# Association between awareness and knowledge of medication-overuse headache with medication-taking behavior among adults with migraine

**DOI:** 10.1371/journal.pone.0306264

**Published:** 2024-06-28

**Authors:** Stacy C. Bailey, Allison P. Pack, Andrea Zuleta, Wei Huang, Melissa P. Herman, Steven M. Kymes, Damian Fiore, Yvonne Curran

**Affiliations:** 1 Division of General Internal Medicine, Feinberg School of Medicine, Northwestern University, Chicago, Illinois, United States of America; 2 H. Lundbeck A/S, Copenhagen, Denmark; 3 Lundbeck LLC, Deerfield, Illinois, United States of America; 4 Department of Neurology, Feinberg School of Medicine, Northwestern University, Chicago, Illinois, United States of America; University of Hafr Al-Batin, SAUDI ARABIA

## Abstract

Frequent use of pain relief medications among patients with migraine can result in disease worsening and medication-overuse headache (MOH), a painful and debilitating condition. We sought to conduct a cross-sectional survey among adult patients diagnosed with migraine to determine: 1) their awareness of MOH, and 2) their knowledge of the condition and its prevention, and 3) the association of these factors with actual use of pain relief medications. We recruited and interviewed 200 English-speaking adults with migraine who had a clinic visit with a neurologist or primary care provider within the past month. Patients were identified via an electronic health record query. Almost 40% of participants had never heard of the term ‘medication-overuse headache.’ In bivariate analyses, participants who were Black or Hispanic and those with limited health literacy were less likely to have heard of MOH. Participants scored an average of 2.1 (range: 0–3) on a MOH knowledge measure; older participants, those with limited health literacy, lower education, and little or no migraine-related disability demonstrated less knowledge. Almost a third (31.5%) of patients reported overusing pain relief medication and were at risk for MOH. Overuse was not significantly associated with MOH awareness, knowledge, or sociodemographic factors, but was related to greater migraine-related disability. Our findings suggest that patient awareness and knowledge of MOH is suboptimal, particularly among older adults, racial and ethnic minority groups, and those with limited health literacy. Interventions are needed to prevent MOH and better inform patients about risks associated with frequent use of pain relief medications.

## Introduction

Medication-overuse headache (MOH), also known as rebound headache, is a common, yet often under-recognized neurologic disorder [1]. MOH occurs when patients with a pre-existing headache disorder take pain relief medication in an attempt to reduce headache pain. Unfortunately, frequent medication use often creates a ‘vicious cycle’ wherein treatment becomes increasingly ineffective for patients, which leads to more headache days per month and a greater need for pain relief. As such, MOH is a common root cause of the transition from episodic to chronic migraine [1]. The condition has been linked to poorer quality of life, greater distress, and worse disability [2, 3]; estimates indicate it could affect half or more of patients with chronic migraine [2, 3].

What constitutes ‘overuse’ varies but is generally defined as taking a pain relief medication on 10–15 or more days each month, depending upon the type of medication [4]. As MOH is largely considered preventable; helping patients to avoid overuse of certain medications can help reduce risk of acquiring the condition [1, 5]. Patient education and counseling is therefore recommended to inform patients of the risks associated with MOH and to ensure that migraine pain relievers—both prescription and over the counter (OTC)—are being used appropriately and effectively [5, 6]. Little is known, however, about migraine patients’ awareness and knowledge of MOH and whether these factors are associated with actual medication use.

To address this gap in the scientific literature, we conducted a cross-sectional, structured survey among adult patients with a diagnosis of migraine receiving care from one large academic health center. We sought to determine: 1) whether patients had heard of MOH, and 2) their knowledge of the condition and how it can be prevented. In parallel, we explored patient-level sociodemographic and clinical factors associated with patients’ MOH awareness and knowledge as well as actual medication use. The study was designed to provide findings to inform future intervention efforts to prevent MOH, particularly among at-risk patient populations.

## Materials and methods

This cross-sectional study collected data from May-October 2022. All research activities were reviewed and approved by the Northwestern University Institutional Review Board (IRB).

### Study participants

The sample for this study consisted of patients from neurology clinics affiliated with one large, academic health center in Chicago, IL and the surrounding suburban areas. To be eligible, patients had to: 1) be age 18 or older, 2) speak English, 3) have a diagnosis of migraine in their electronic health record (EHR), 4) have an appointment with a neurology provider within the past month for migraine, 5) have access to a phone or the internet, 5) have an active email account, 6) have access to video conferencing technology, and 7) have a mobile phone with text messaging capability. Patients with any severe, uncorrectable vision, hearing, or cognitive impairment that would preclude study participation or consent were excluded from the study.

### Recruitment

Potential participants were initially identified via an EHR query and sent letters describing the study. Letters included an option to email or call the research team to ‘opt out’ of being contacted. Those who did not opt out were then contacted by phone, screened for eligibility, and engaged in the IRB-approved informed consent process by a research coordinator. Specifically, to obtain verbal consent, the coordinator read a study consent form aloud to the participant, pausing frequently to allow opportunities for clarification, and elicited verbal consent. The coordinator digitally signed their own name on an electronic consent form; this was saved on a secure server. Participants were then mailed or emailed a copy of the consent form for their records.

### Data collection procedures and measures

Research staff completed a structured survey with enrolled participants via a videoconferencing technology platform; participant responses were recorded in REDCap. Surveys took approximately 45 minutes to complete and participants were given $40 for their time.

Surveys included measures of participant sociodemographic characteristics (age, sex, race, ethnicity, income level, education). Health literacy was measured via the Health Literacy-6 (HL6), with participants classified as having either limited or adequate health literacy [7]. Migraine-related disability was measured according to the validated Migraine Disability Assessment Questionnaire (MIDAS) [7]. Participants were categorized according to published criteria as having little or no disability, mild or moderate disability, or severe disability [8].

MOH awareness was assessed by asking participants whether they had ever heard of the term ‘medication-overuse headache’ (yes/no). MOH knowledge was assessed by asking participants: 1) whether medications that are used to treat headaches can also cause headaches (true/false), 2) whether certain medications (e.g. combination pain relievers, acetaminophen, aspirin) can cause MOH (yes/no, per medication type; all had to be answered correctly to receive credit), and 3) how often OTC medications can be taken to avoid MOH (every time you have a headache vs. two times per week or less). The number of correct answers was summed to create a knowledge score of 0 to 3, with higher scores indicating greater knowledge.

Participants were also asked to report all OTC and prescription medications taken over the past month and the frequency of use for each. Taking combination pain relievers, triptans, ergotamines or opioids 10 or more days per month, or simple analgesics 15 or more days per month, was categorized as ‘medication overuse’ in accordance with clinical guidance [4].

### Analyses

Simple descriptive statistics were calculated for variables measuring patients’ sociodemographic and migraine clinical characteristics. Summary statistics were used to describe patient outcomes, including awareness and knowledge of MOH. Bivariate analyses, utilizing either χ^2^, t tests, Pearson correlation tests, or analysis of variance (ANOVA) as appropriate, were conducted to examine differences in MOH awareness and knowledge by participant sociodemographic characteristics, health literacy, and migraine-related disability. Similar analyses were performed to examine medication overuse and its relationship to MOH awareness, knowledge and patient characteristics. All quantitative analyses were performed using SAS software (version 9.4, Cary, NC).

## Results

### Study sample

A total of 874 participants were contacted for this study. Of those, 40 could not be reached, 147 refused to participate, 480 were found to be ineligible during the screening process, 7 could not be scheduled for an interview or withdrew before the interview, and 200 were enrolled. Patients were predominately female (82.9%) and on average were 43 years old; about half (57.4%) were White, 15.9% were Hispanic and 14.9% were Black. About one-third (31.8%) made less than $50,000 in household income annually and almost 1 in 5 (18.2%) had limited health literacy according to the HL6. Nearly two-thirds of participants (64.0%) were classified as having severe migraine-related disability. Patients had been diagnosed with migraine an average of 17.8 years (Table 1).

**Table 1:** Migraine overuse headache awareness and knowledge by participant characteristics.

Participant Characteristic	Overall (N = 200)	Awareness, N(%)		P-value	Knowledge Mean(SD) (N = 200)	P-value
		Yes (N = 121)	No (N = 79)			
Sex^a^				0.34		0.55
Male	34(17.1)	18(15.0)	16(20.3)		2.2(0.9)	
Female	165(82.9)	102(85.0)	63(79.8)		2.1(0.9)	
Age				0.09		<0.001*
18–30	45(22.5)	32(26.5)	13(16.5)		2.5(0.6)	
31–50	91(45.5)	48(39.7)	43(54.4)		2.1(0.9)	
>50	64(32.0)	41(33.9)	23(29.1)		1.8(0.9)	
Race^b^				0.009*		0.15
Hispanic	31(15.9)	13(10.8)	18(24.0)		1.9(0.8)	
Black	29(14.9)	15(12.5)	14(18.7)		1.8(1.0)	
White	112(57.4)	80(66.7)	32(42.7)		2.2(0.9)	
Other	23(11.8)	12(10.0)	11(14.7)		2.2(0.8)	
Education				0.16		0.02*
High school graduate or less	26(13.0)	13(10.7)	13(16.5)		1.7(1.0)	
Some college or technical school	44(22.0)	28(23.1)	16(20.3)		2.1(0.9)	
College graduate	64(32.0)	34(28.1)	30(38.0)		2.0(0.9)	
Graduate degree or more	66(33.0)	46(38.0)	20(25.3)		2.9(0.8)	
Income^c^				0.24		0.44
Less than $25,000	31(17.3)	21(19.8)	10(13.7)		2.1(0.8)	
$25,000 - $49,999	26(14.5)	11(10.4)	15(20.6)		1.9(1.0)	
$50,000 - $99,999	44(24.6)	25(23.6)	19(26.0)		2.3(0.9)	
$100,000 - $199,000	40(22.4)	23(21.7)	17(23.3)		2.0(0.8)	
$200,000 or more	38(21.2)	26(24.5)	12(16.4)		2.9(0.8)	
Health literacy^d^				0.02*		0.01*
Limited	34(18.2)	15(13.0)	19(26.4)		1.7(1.0)	
Adequate	153(81.8)	100(87.0)	53(73.6)		2.1(0.8)	
Migraine-related disability				0.18		0.03*
Little or no disability	27(13.5)	12(9.9)	15(19.0)		1.7(0.9)	
Mild or moderate disability	45(22.5)	29(24.0)	16(20.3)		2.2(0.8)	
Severe disability	128(64.0)	80(66.1)	48(60.8)		2.1(0.9)	
Years since migraine diagnosis, mean(SD)	17.8(13.9)	18.8(14.0)	16.2(13.7)	0.20	-	0.77

a.1 missing

b. 5 missing

c. 21 missing

d. 13 missing

*Statistically significant at p≤.05

### Medication-overuse headache awareness

More than one-third of respondents (39.5%) reported they had never heard the term ‘medication-overuse headache.’ In bivariate analyses, participants who were Black or Hispanic were significantly less likely than White patients to have heard of MOH. Participants with limited health literacy were also less likely to have heard of MOH than those with adequate (Table 1).

### Medication-overuse headache knowledge

Almost 1 in 5 participants (19.0%) did not know that ‘medications that are taken to treat headache can also cause headaches.’ Similarly, less than half (41.0%) of participants were able to correctly identify medications that can cause MOH (e.g., combination pain relievers, aspirin, acetaminophen). Finally, 15.0% of participants incorrectly endorsed that OTC medications can be taken ‘every day you have a headache’ to avoid MOH. When items were summed into a knowledge score, the average score was 2.1 (range 0 to 3, with higher scores indicating greater knowledge). Participants who were older, those with limited health literacy, adults with a high school or lower level of education, and patients with little or no migraine-related disability were significantly less knowledgeable about MOH than their counterparts (Table 1).

### Medication overuse

Most participants (84.5%) reported taking a prescription medication to prevent or treat migraine and two thirds (66.0%) said they took OTC medication for migraine. Almost a third of all participants (31.5%) reported taking either a prescription or OTC medication at a frequency that qualified as overuse. Neither MOH awareness nor knowledge were significantly associated with medication overuse. Only migraine-related disability was significantly related to medication-taking behavior; patients with more severe migraine-related disability were more likely to take medications at a higher frequency than recommended (Table 2).

**Table 2:** Medication overuse, by participant characteristics, awareness and knowledge.

Participant Characteristics	Overall (N = 200)	Medication Overuse		P-value
		No (N = 137)	Yes (N = 63)	
Sex^a^, n(%)				0.13
Male	34(17.1)	27(19.9)	7(11.1)	
Female	165(82.9)	109(80.2)	56(88.9)	
Age, n(%)				0.51
18–30	45(22.5)	30(21.9)	15(23.8)	
31–50	91(45.5)	66(48.2)	25(39.7)	
>50	64(32.0)	41(29.9)	23(36.5)	
Race and ethnicity^b^, n(%)				0.95
Hispanic	31(15.9)	21(15.8)	10(16.1)	
Non-Hispanic Black	29(14.9)	21(15.8)	8(12.9)	
Non-Hispanic White	112(57.4)	75(56.4)	37(59.7)	
Other	23(11.8)	16(12.0)	7(11.3)	
Education, n(%)				0.85
Some college or less	70(35.0)	47(34.3)	23(36.5)	
College graduate	64(32.0)	43(31.4)	21(33.3)	
Graduate degree or more	66(33.0)	47(34.3)	19(30.2)	
Income^c^, n (%)				0.11
< $50,000	57(31.8)	38(30.7)	19(34.6)	
$50,000- $99,999	44(24.6)	26(21.0)	18(32.7)	
$100,000 or more	78(43.6)	60(48.4)	18(32.7)	
Health literacy^d^, n(%)				0.07
Limited	34(18.2)	28(21.5)	6(10.5)	
Adequate	153(81.8)	102(78.5)	51(89.5)	
Migraine-related disability, n(%)*				0.01
Little or no disability	27(13.5)	20(14.6)	7(11.1)	
Mild or moderate disability	45(22.5)	39(28.5)	6(9.5)	
Severe disability	128(64.0)	78(56.9)	50(79.4)	
MOH Awareness, n(%)	121(60.5)	78(56.9)	43(68.3)	0.13
MOH Knowledge, mean(SD)	2.1(0.9)	2.2(0.9)	1.9(0.9)	0.07
Years since migraine diagnosis, mean(SD)	17.8(13.9)	16.7(13.5)	20.1(14.6)	0.10

a. 1 missing

b. 5 missing

c. 21 missing

d. 13 missing

*Statistically significant at p≤.05

## Discussion

Findings indicate MOH awareness and knowledge are suboptimal among patients with migraine. More than one-third of participants in this study reported that they had never heard of the term ‘medication-overuse headache’ despite being diagnosed with migraine for an average of 17.8 years. In parallel, 1 in 5 patients did not know that medications taken to treat headaches can also cause headaches. They also displayed limited understanding of which medications can cause MOH and how often medications can be taken to avoid the condition. In particular, older adults, individuals with a high school or lower level of education, and those with limited health literacy were less aware and less knowledgeable about MOH. This patient population is likely to need additional education and counseling on MOH and appropriate medication use. Providers should ensure that education and counseling incorporate evidence-based health literacy ‘best practices’ to promote learning and help bridge this divide [9, 10].

Nearly a third of participants in our study reported taking prescription or OTC medication at a frequency that places them at risk for MOH. Yet there were no significant relationships found between medication overuse and MOH awareness, knowledge, or any patient sociodemographic characteristics. The only factor related to medication overuse was migraine-related disability, with patients who reported more severe disability being more likely to overuse medication. This may reflect patient challenges controlling migraine pain and symptoms and the ‘vicious cycle’ of medication overuse that can lead to more frequent headaches and greater disability. While it is plausible that knowledge alone may be insufficient to prevent high-risk medication use among patients experiencing high levels of pain and disability due to migraine, additional studies will be needed to examine and elucidate this relationship fully.

We are unaware of any other studies conducted in the United States that have sought to examine patient awareness and knowledge of MOH and the relationship of these factors to medication overuse; likewise, a research gap exists on strategies to reduce medication overuse in this country. However, several small intervention trials conducted in Europe have reported a link between MOH knowledge and medication use, specifically among patients already diagnosed with MOH. These studies found that providing simple information or advice about MOH reduced overall medication use and days with headache, along with other outcomes [11–14]. Similarly, public awareness campaigns in Japan, Norway, Russia, and Denmark have sought to inform the general public about MOH and headache management [15–18]. These campaigns have used a variety of strategies–including leaflets, mass media, e-learning modules, and online resources–and experienced variable, yet often marginal, improvements in MOH-related outcomes. While lessons can be learned from these efforts, there may be cultural and/or health system factors specific to the United States that affect how migraine patients access and take medication in this country. Additional research is therefore needed to identify optimal approaches to improving medication use among patients with migraine in the United States.

There are limitations to this study that should be noted. First, participants were recruited from one large academic health center in the Midwest region of the United States. Thus, results may not be generalizable to other regions, health systems, or patient populations. However, the characteristics of our study sample are broadly reflective of the population of patients diagnosed with migraine in the United States, which is predominately female, approaching middle age, and White [19, 20]. Secondly, this study was cross-sectional in design. We therefore cannot make any inferences about causation and can only examine associations between factors at one time point. Third, while our survey had an adequate response rate, it was conducted among a relatively small sample of participants and not all eligible patients chose to participate; this may have introduced bias. Finally, the MOH knowledge items were created by our team as no validated questionnaires on this topic could be found. Additional studies using robust measures among larger patient populations are needed to further explore these relationships and provide more generalizable results.

## Conclusion

In summary, our findings indicate a need for greater patient education and counseling on MOH to increase awareness and knowledge of the condition. Yet our results also indicate that additional support–beyond education–may be needed to help those individuals with greater migraine-related disability avoid overusing medication and worsening their condition. Additional studies are needed to confirm our findings and to determine what types of support could be beneficial for patients with, and at risk of, MOH.
